# Improving quality of stroke care through benchmarking center performance: why focusing on outcomes is not enough

**DOI:** 10.1186/s12913-020-05841-y

**Published:** 2020-10-31

**Authors:** Marzyeh Amini, Nikki van Leeuwen, Frank Eijkenaar, Maxim J. H. L. Mulder, Wouter Schonewille, Geert Lycklama à Nijeholt, Wouter H. Hinsenveld, Robert-Jan B. Goldhoorn, Pieter Jan van Doormaal, Sjoerd Jenniskens, Jan Hazelzet, Diederik W. J. Dippel, Bob Roozenbeek, Hester F. Lingsma, on behalf of the MR CLEAN Registry Investigators, Diederik W. J. Dippel, Aad van der Lugt, Charles B. L. M. Majoie, Yvo B. W. E. M. Roos, Robert J. van Oostenbrugge, Wim H. van Zwam, Jelis Boiten, Jan Albert Vos, Josje Brouwer, Sanne J. den Hartog, Wouter H. Hinsenveld, Manon Kappelhof, Kars C. J. Compagne, Robert-Jan B. Goldhoorn, Maxim J. H. L. Mulder, Ivo G. H. Jansen, Bob Roozenbeek, Adriaan C. G. M. van Es, Bart J. Emmer, Jonathan M. Coutinho, Wouter J. Schonewille, Marieke J. H. Wermer, Marianne A. A. van Walderveen, Julie Staals, Jeannette Hofmeijer, Jasper M. Martens, Geert J. Lycklama à Nijeholt, Sebastiaan F. de Bruijn, Lukas C. van Dijk, H. Bart van der Worp, Rob H. Lo, Ewoud J. van Dijk, Hieronymus D. Boogaarts, J. de Vries, Paul L. M. de Kort, Julia van Tuijl, Jo Jo P. Peluso, Puck Fransen, Jan S. P. van den Berg, Boudewijn A. A. M. van Hasselt, Leo A. M. Aerden, René J. Dallinga, Maarten Uyttenboogaart, Omid Eschgi, Reinoud P. H. Bokkers, Tobien H. C. M. L. Schreuder, Roel J. J. Heijboer, Koos Keizer, Lonneke S. F. Yo, Heleen M. den Hertog, Emiel J. C. Sturm, Paul Brouwers, Marieke E. S. Sprengers, Sjoerd F. M. Jenniskens, René van den Berg, Albert J. Yoo, Ludo F. M. Beenen, Alida A. Postma, Stefan D. Roosendaal, Bas F. W. van der Kallen, Ido R. van den Wijngaard, Joost Bot, Pieter-Jan van Doormaal, Anton Meijer, Elyas Ghariq, Marc P. van Proosdij, G. Menno Krietemeijer, Jo P. Peluso, Rob Lo, Dick Gerrits, Wouter Dinkelaar, Auke P. A. Appelman, Bas Hammer, Sjoert Pegge, Anouk van der Hoorn, Saman Vinke, H. Zwenneke Flach, Hester F. Lingsma, Naziha el Ghannouti, Martin Sterrenberg, Corina Puppels, Wilma Pellikaan, Rita Sprengers, Marjan Elfrink, Michelle Simons, Marjolein Vossers, Joke de Meris, Tamara Vermeulen, Annet Geerlings, Gina van Vemde, Tiny Simons, Cathelijn van Rijswijk, Gert Messchendorp, Nynke Nicolaij, Hester Bongenaar, Karin Bodde, Sandra Kleijn, Jasmijn Lodico, Hanneke Droste, Maureen Wollaert, Sabrina Verheesen, D. Jeurrissen, Erna Bos, Yvonne Drabbe, Michelle Sandiman, Nicoline Aaldering, Berber Zweedijk, Mostafa Khalilzada, Jocova Vervoort, Eva Ponjee, Sharon Romviel, Karin Kanselaar, Denn Barning, Esmee Venema, Vicky Chalos, Ralph R. Geuskens, Tim van Straaten, Saliha Ergezen, Roger R. M. Harmsma, Daan Muijres, Anouk de Jong, Olvert A. Berkhemer, Anna M. M. Boers, J. Huguet, P. F. C. Groot, Marieke A. Mens, Katinka R. van Kranendonk, Kilian M. Treurniet, Ivo G. H. Jansen, Manon L. Tolhuisen, Heitor Alves, Annick J. Weterings, Eleonora L. F. Kirkels, Eva J. H. F. Voogd, Lieve M. Schupp, Sabine Collette, Adrien E. D. Groot, Natalie E. LeCouffe, Praneeta R. Konduri, Haryadi Prasetya, Nerea Arrarte-Terreros, Lucas A. Ramos

**Affiliations:** 1 Department of Public Health, Erasmus MC University Medical Center, P.O. Box 2040, 3000 CA Rotterdam, The Netherlands; 2 Erasmus School of Health Policy & Management, Erasmus University Rotterdam, Rotterdam, The Netherlands; 3 Department of Neurology, Erasmus MC University Medical Center, Rotterdam, The Netherlands; 4 Department of Neurology, St. Antonius Hospital, Nieuwegein, The Netherlands; 5 Department of Radiology, Medical Center Haaglanden, The Hague, The Netherlands; 6 Maastricht University Medical Center and Cardiovascular Research Institute Maastricht (CARIM), Maastricht, The Netherlands; 7 Department of Radiology and Nuclear Medicine, Erasmus MC University Medical Center, Rotterdam, The Netherlands; 8 Department of Radiology, Radboud University Medical Center, Nijmegen, The Netherlands

**Keywords:** Stroke, Endovascular treatment, Benchmarking, Quality of care, Outcome differences, Case-mix, Process of care

## Abstract

**Background:**

Between-center variation in outcome may offer opportunities to identify variation in quality of care. By intervening on these quality differences, patient outcomes may be improved. However, whether observed differences in outcome reflect the true quality improvement potential is not known for many diseases. Therefore, we aimed to analyze the effect of differences in performance on structure and processes of care, and case-mix on between-center differences in outcome after endovascular treatment (EVT) for ischemic stroke.

**Methods:**

In this observational cohort study, ischemic stroke patients who received EVT between 2014 and 2017 in all 17 Dutch EVT-centers were included. Primary outcome was the modified Rankin Scale, ranging from 0 (no symptoms) to 6 (death), at 90 days. We used random effect proportional odds regression modelling, to analyze the effect of differences in structure indicators (center volume and year of admission), process indicators (time to treatment and use of general anesthesia) and case-mix, by tracking changes in *tau*^2^, which represents the amount of between-center variation in outcome.

**Results:**

Three thousand two hundred seventy-nine patients were included. Performance on structure and process indicators varied significantly between EVT-centers (*P* < 0.001). Predicted probability of good functional outcome (modified Rankin Scale 0–2 at 90 days), which can be interpreted as an overall measure of a center’s case-mix, varied significantly between 17 and 50% across centers. The amount of between-center variation (*tau*^*2*^) was estimated at 0.040 in a model only accounting for random variation. This estimate more than doubled after adding case-mix variables (*tau*^2^: 0.086) to the model, while a small amount of between-center variation was explained by variation in performance on structure and process indicators (*tau*^2^: 0.081 and 0.089, respectively). This indicates that variation in case-mix affects the differences in outcome to a much larger extent.

**Conclusions:**

Between-center variation in outcome of ischemic stroke patients mostly reflects differences in case-mix, rather than differences in structure or process of care. Since the latter two capture the real quality improvement potential, these should be used as indicators for comparing center performance. Especially when a strong association exists between those indicators and outcome, as is the case for time to treatment in ischemic stroke.

**Supplementary Information:**

The online version contains supplementary material available at 10.1186/s12913-020-05841-y.

## Background

Endovascular treatment (EVT) has been shown to be a highly effective treatment for patients with ischemic stroke due to a proximal intracranial occlusion in the anterior circulation [1–3]. EVT is defined as arterial catheterization with a micro-catheter to the level of the occlusion, followed by mechanical thrombectomy or thrombus aspiration, or both, with or without delivery of a thrombolytic agent. Currently EVT is widely implemented in routine clinical practice, and the challenge is how to continuously improve the quality of this service.

Worldwide, healthcare systems and practices are being reorganized with a strong focus on measuring and improving outcomes of care. The quality of care for patients with a specific medical condition is judged by the achieved outcomes that are relevant for those patients. A central aspect of this development is benchmarking, comparing quality of care and specifically outcomes between healthcare providers, in this case EVT centers. If a specific center A has better outcomes for a certain condition than center B, this suggests that center B should copy the medical management strategy of center A in order to improve the quality of care in that center. An important problem of this approach, however, is the variability in baseline characteristics of patients (‘case-mix’) that often exists between centers. If center B treats more severely affected patients than center A, this might (partly) explain the better outcomes of center A. Moreover, especially if centers are relatively small, between-center differences in outcome may be caused by chance (‘random variation’). Therefore, between-center comparisons of outcome should be adjusted for case-mix and random variation. If not done properly, such comparisons are likely to miss their purpose and could even be counterproductive as clinicians may base their decisions on flawed information. Furthermore, following Donabedian’s framework for evaluating healthcare quality, the quality improvement potential is especially captured by variation in structures (‘How is care organized?’) and processes (‘What is done?’) of care [4]. For many diseases, however, it is unknown whether between-center variation in outcome reflects true differences in quality of care, captured by this framework.

Using data from a large nation-wide registry, the aim of this study was to assess the effect of structure and process indicators on between-center variation in outcome for ischemic stroke patient treated with EVT, while adjusting for case-mix and random variation.

## Methods

### Study design and patients

For this study we used data collected between March 2014 and November 2017 from the MR CLEAN Registry, a prospective, observational study in all 17 centers that perform EVT in the Netherlands (Supplementary Figure 1) [5]. This registry is unique since it includes clinical and neuro-imaging data of all patients treated with EVT in one country during a multi-year period and thereby reflects clinical practice. All patients undergoing EVT for acute ischemic stroke have been registered. Inclusion criteria were: age 18 years and older, treatment in a center that participated in the MR CLEAN trial, and proximal intracranial vessel occlusion in the anterior circulation (internal carotid artery (ICA), internal carotid artery terminus (ICA-T), middle (M1/M2) cerebral artery, or anterior (A1/A2) cerebral artery), as shown by computed tomography angiography (CTA). Details on the study design and objectives of the MR CLEAN Registry have been described elsewhere [5]. Overall, data from 3279 patients were included for the current analysis (Supplementary Figure 2).

The MR CLEAN Registry was approved by the ethics committee of the Erasmus University MC, Rotterdam, The Netherlands (MEC-2014-235). With this approval it was approved by the research board of each participating center. At UMC Utrecht, approval to participate in the study has been obtained from their own research board and ethics committee.

### Case-mix indicators

For case-mix adjustment we used the following patient and neuro-imaging characteristics: age, sex, relevant medical history (i.e. previous stroke, atrial fibrillation, myocardial infarction, peripheral arterial disease, hypertension, diabetes mellitus, hypercholesterolemia), pre-stroke score on the modified Rankin Scale (mRS), the baseline score on the National Institute of Health Stroke Scale (NIHSS) as a stroke-related neurologic deficit score, and occlusion location and collateral grade on CT angiography. These characteristics were selected based on clinical knowledge and previous studies [6, 7]. Two additional characteristics were considered as case-mix indicators, because these are strongly associated with outcome but not influenceable by the centers: time between stroke onset and arrival at the emergency department (ED) of the intervention center, and whether or not the patient had been admitted to another center before being transferred to the intervention center [8, 9]. Stroke onset was defined as the time point when the sudden appearance of stroke symptoms was witnessed by the patient or an observer. In cases the time of first symptoms was unknown, onset was defined as the moment the patient was last seen well.

### Quality of care indicators

Quality of care indicators were defined using Donabedian’s framework comprising indicators of structure, process, and outcome [4]. Both center volume and year of admission reflect the experience of a center with EVT and were used as structure indicators. Center volume was defined as the percentage of all EVT-patients treated in each center relative to all EVT-patients treated in the Netherlands in the study period. In stroke care, high center volume was found to be associated with lower stroke-related mortality [10]. In other studies, higher volume stroke centers showed better outcomes on average [11–13]. Since EVT is a relatively new treatment in stroke care, we hypothesized (overall) performance to increase with calendar year and therefore added year of admission as an additional structure indicator.

Two process indicators were defined: time from arrival at the emergency department of the intervention center to groin puncture and the use of general anesthesia (yes/no). A significant negative association between ‘time-to-groin’ and outcome was found in previously published research, indicating that time delays before initiation of EVT have a negative effect on the likelihood of independent functional recovery at 90 days [8, 13–17]. So far, no differences in outcomes between general anesthesia and conscious sedation were observed in previous randomized controlled trials [18–20]. In several meta-analyses of observational studies, conscious sedation was associated with better outcomes than general anesthesia [21–23]. Although general anesthesia reduces the risk of patient agitation, unnecessary use of general anesthesia increases time delay in the total process of care, intra-procedural complications, and may result in cerebral hypoperfusion (e.g. through fluctuations in blood pressure on general anesthesia induction and abnormal cerebral auto-regulation) [24, 25]. Overall, we expected the use of general anesthesia to influence patient outcome, and therefore defined this as an additional process indicator.

We used the modified Rankin Scale (mRS) score as the outcome indicator [26]. The mRS score is a commonly used measure of patients’ functional outcome after ischemic stroke, and ranges from 0 (no symptoms) to 6 (death). The mRS score was assessed at 90 days after EVT (± 14 days). Good functional outcome, defined as mRS 0–2, was used as secondary outcome (see below).

### Statistical analyses

#### Descriptive analyses

We used Pearson’s chi-square statistic and the non-parametric Kruskal Wallis test for a univariable comparison of centers on case-mix and quality of care indicators. The predicted probability of good functional outcome can be considered an overall measure of each centers’ case-mix. To calculate this, we first fitted an individual-level logistic regression model including all case-mix indicators as predictors and yes/no good functional outcome (mRS 0–2 at 90 days) as the dependent variable. The predicted patient-level probabilities by this model were then used to calculate the median predicted probability per center.

#### Random effect regression models

In order to adjust for random variation and assess the effects of adjusting for case-mix and performance on structure and process indicators on between-center variation in outcome, we used random effect proportional odds regression modelling. A random center effect (intercept) accounts for the fact that the observed outcomes for lower-volume centers can take extreme values due to random variation. A proportional odds model exploits the full ordinal nature of the mRS as an outcome scale with more than two possible categories [27].

In all analyses, we used the inverse of the mRS score for each patient. Doing so allows us to interpret the estimates as the effects on the likelihood of a more favorable outcome, since a higher score on the inverse of the mRS means more favorable outcome (see above). We estimated common odds ratios with 95% confidence intervals on the patient level using four proportional odds regression models [27]. First, we fitted an ‘empty’, unadjusted model including only a random center effect, providing insight in between-center variation in outcome accounting only for random variation. In the second model, in addition to the random center effect, we adjusted for the individual-level fixed effects of case-mix indicators on outcome. In the third model, we added the fixed effects of the structure indicators to the model. Finally, the fourth model also contains the individual-level fixed effects of the process indicators.

#### Between-center variation

To assess the relative impact of adjusting for case-mix and performance on structure and process indicators on between-center variation in outcome, we compared the variance of the random center effect (*tau*^2^) across models. Essentially, *tau*^2^ reflects the amount of between-center variation in outcome. In addition, separately for each model, we constructed forest plots to visualize this variation using estimates of center-specific outcome (i.e. the random center effects). The predictive power of the four models was compared using Akaike’s Information Criterion (AIC), in which a lower AIC value indicates a higher predictive power for outcome [28].

#### Missing data

Multiple imputation was used to deal with missing data, which ranged between 0.7% (previous diabetes) to 6.3% (collateral grade) [5]. We fitted regression imputation models [29, 30] and imputed data five times, using the following variables: age, sex, medical history, pre-stroke mRS score, location of occlusion, collateral grade, baseline NIHSS score, whether patient transferred from other hospital, time intervals from onset to arrival at the ED, center volume per year, year of admission, time intervals from onset to groin puncture, and use of general anesthesia. Each imputed dataset was analyzed separately, after which the results were pooled.

All statistical analyses were performed with R statistical software version 3.4.3 (R Foundation for Statistical Computation, Vienna, Austria), using the *clmm* module in the *ordinalimputation* package. Statistical significance was assessed at *P* < 0.05 in all analyses.

## Results

### Descriptive analyses

At the center level, the median patient age ranged from 68 to 77 years, with statistically significant differences between centers (Table 1). Differences between centers were also statistically significant for previous stroke (range 0–26%), atrial fibrillation (13–37%), peripheral arterial disease (4–22%), hypertension (41–67%), hypercholesterolemia (15–50%), pre-stroke mRS, location of occlusion, collateral grade, baseline NIHSS score (range median score per center 13–17) and percentage of transferred patients from another hospital (0–77%). Median time of stroke onset to arrival at the ED of the intervention center ranged from 52 to 160 min across centers (*P* < 0.001). Importantly, the median predicted probability of good functional outcome (mRS 0–2 at 90 days), which can be interpreted as an overall measure of a center’s case-mix, varied between 17 and 50% across centers (*P* = 0.004) (Table 1 and Supplementary Table 1). The patient-level effect estimates of case-mix variables on outcome shown in Supplementary Figure 3 are comparable to the results of prior research [31].

**Table 1:** Case-mix characteristics of patients treated in intervention centers in MR CLEAN Registry

	N (%) / Median (IQR) in total population (missing excluded)	Center-level range (median or percentage)	Total number of patients	Missing N (%)	P-value*
Age (years)	72 (61–80)	68–77	3279	0	0.001
Men	1696 (52)	39–55	3279	0	0.790
Medical History					
Previous Stroke	546 (17)	0–26	3252	27 (0.8)	< 0.001
Atrial Fibrillation	772 (24)	13–37	3236	43 (1.3)	< 0.001
Myocardial Infarction	453 (14)	4–18	3212	67 (2.0)	0.16
Peripheral Arterial Disease	301 (9)	4–22	3211	68 (2.1)	< 0.001
Hypertension	1688 (53)	41–67	3213	66 (2.0)	< 0.001
Diabetes	532 (16)	12–25	3255	24 (0.7)	0.09
Hypercholesterolemia	967 (31)	15–50	3136	143 (4.4)	< 0.001
Pre-stroke modified Rankin Scale score			3207	72 (2.2)	< 0.001
0	2170 (68)	45–87			
1	424 (13)	3–19			
2	241 (8)	0–16			
≥ 3	372 (12)	5–25			
Location of occlusion			3119	160 (4.99)	< 0.001
M1	1815 (58)	45–70			
M2	455 (15)	4–31			
Intracranial ICA	161 (5)	0–10			
ICA-T	663 (21)	9–27			
Other (M3/anterior)	25 (1)	0–4			
Collateral grade			3072	207 (6.3)	< 0.001
0 (Absent)	187 (6)	0–9			
1 (< 50%)	1100 (36)	17–46			
2 (> 50% but < 100%)	1190 (39)	33–61			
3 (100%)	595 (19)	10–37			
Baseline NIHSS score	16 (11–20)	13–17	3224	55 (1.7)	< 0.001
Transferred patients from other hospital	1783 (54)	0–77	3279	0	< 0.001
Time from onset to arrival at the ED (min)	135 (65–195)	52–160	3155	124 (3.8)	< 0.001
Predicted probability of good functional outcome^a^	41 (20–62)	17–50	2550	729 (22.2)	0.004

*IQR* Interquartile range, *ICA-T* internal carotid artery terminus, *M1/M2* middle cerebral artery, *NIHSS* National Institutes of Health Stroke Scale, *ED* Emergency department

^a^Predicted probability (%) of good functional outcome (modified Rankin Scale score 0–2 at 90 days) is based on an individual-level logistic regression model predicting good functional outcome from the case-mix variables included in this table. See Supplementary Table 1 for details of case-mix characteristics of patients treated in each intervention center

*P-value is based on comparison between 17 centers using a non-parametric Kruskal Wallis test for continuous variables or Pearson’s chi-square statistic for categorical variables

**Fig. 1:**
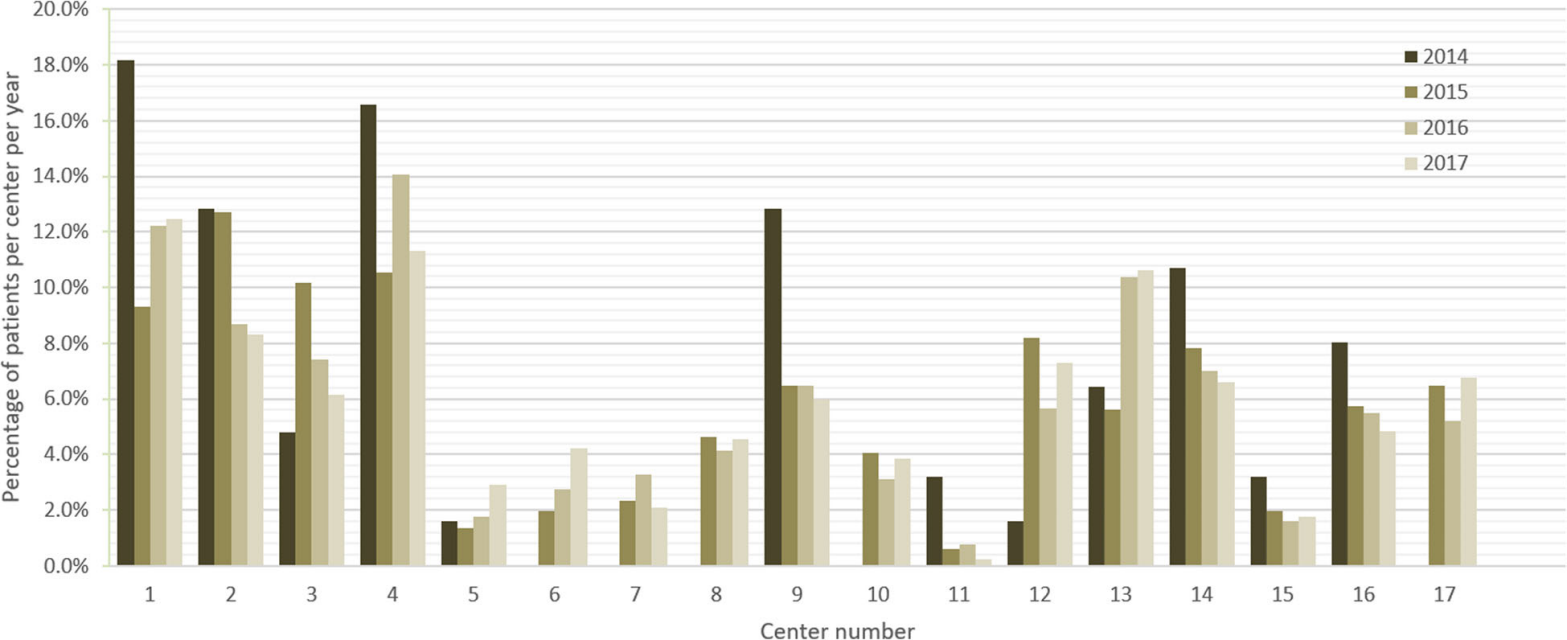
Center volume in each intervention year. Center volume is defined as percentage of all EVT patients treated in each center relative to all EVT patients treated in the Netherlands in that year

Relative to the total number of patients in our data, the number of EVT-patients varied significantly between centers across the 4 years (Fig. 1). Median time from arrival at the ED of the intervention center to groin puncture also varied substantially: between 74 and 125 min for non-transferred patients (*P* < 0.001) and between 20 and 60 min for transferred patients (*P* < 0.001). Variation in the use of general anesthesia (0–99%) was also statistically significant. Crude differences in outcome were statistically significant (*P* < 0.001) across centers for mRS values 0–6: no symptoms (1–18%), no significant disability (7–26%), slight disability (4–29%), moderate disability (4–20%), moderately severe disability (7–31%), severe disability (2–11%), and death (21–36%) (Table 2 and Supplementary Table 2).

**Table 2:** Quality of care indicators of all 17 intervention centers in the Netherlands

	N (%) / Median (IQR) in total population (missing excluded)	Center-level range (median or percentage)	Total number of patients	Missing N (%)	P-value*
Structure					
Center volume			3279	0	
2014	187 (6^a^)	0–18			< 0.001
2015	822 (25)	1–13			< 0.001
2016	1131 (34)	1–14			< 0.001
2017	1139 (35)	0.3–13			< 0.001
Processes					
Time from arrival at the ED to groin puncture in non-transferred patients (min)	90 (70–119)	74–125	1292	203 (6.2)	< 0.001
Time from arrival at the ED to groin puncture in transferred patients (min)	39 (26–57)	20–60	1722	61 (1.9)	< 0.001
Use of general anesthesia	778 (25)	0–99	3082	197 (6.0)	< 0.001
Outcome					
Modified Rankin Scale (mRS) score at 90 days			3065	214 (6.5)	< 0.001
mRS 0 (No symptoms)	209 (7)	1–18			
mRS 1 (No significant disability)	471 (15)	7–26			
mRS 2 (Slight disability)	561 (18)	4–29			
mRS 3 (Moderate disability)	404 (13)	4–20			
mRS 4 (Moderately severe disability)	366 (12)	7–31			
mRS 5 (Severe disability)	168 (6)	2–11			
mRS 6 (Dead)	886 (29)	21–36			

*IQR* Interquartile range, *ED* Emergency department, *EVT* endovascular treatment

^a^Percentage of all EVT patients treated in the Netherlands in that year (see Fig. 1 for details)

*P-value is based on comparison between 17 centers using a non-parametric Kruskal Wallis test for continuous variables or Pearson’s chi-square statistic for categorical variables

See Supplementary Table 1 for details of quality of care indicators in each intervention center

### Predictive power for outcome

Model 1 generated an AIC of 11,158, which dropped to 10,050 after adding case-mix variables in model 2, suggesting a considerably improved predictive power (Table 3). Adding structure (AIC = 10,043) and process indicators (AIC = 10,012) only slightly improved the predicted power further (Table 3).

**Table 3:** Results from the random effect proportional odds regression analysis using the inverse of the modified Rankin Scale at 90 days as the dependent variable

	Model 1	Model 2	Model 3	Model 4
Center volume [OR (95% CI)]	–	–	1.01 (0.98–1.04)	1.01 (0.98–1.05)
Year of admission [OR (95% CI)]	–	–		
2014			ref	ref
2015			1.32 (0.95–1.85)	1.20 (0.86–1.67)
2016			1.67 (1.22–2.28)	1.36 (0.98–1.90)
2017			1.60 (1.17–2.19)	1.27 (0.91–1.77)
Time from arrival at the ED of intervention center to groin puncture (every 30 min) [OR (95% CI)]	–	–	–	0.87 (0.82–0.92)
Use of general anesthesia [OR (95% CI)]	–	–	–	0.72 (0.57–0.90)
Variance of random center intercept [*tau*^2^(95% CI)]^#^	0.040 (0.012–0.113)	0.086 (0.042–0.261)	0.081 (0.028–0.227)	0.089 (0.033–0.254)
AIC	11,158	10,050	10,043	10,012

*ED* Emergency department, *AIC* Akaike’s Information Criterion. A lower AIC value indicates a better model fit

Model 1 (‘unadjusted model’) includes a random center intercept only

Model 2 (‘case-mix adjusted model’) includes a random center intercept and the case-mix indicators (Table 1). See Supplementary Figure 3 for the estimated fixed effects of each case-mix indicator

Model 3 (‘case-mix and structure indicators adjusted model’) includes a random center intercept, case-mix indicators, and structure indicators

Model 4 (‘case-mix, structure and process indicators adjusted model’) includes a random center intercept, case-mix indicators, structure indicators, and process indicators

^#^95% CIs around *τau*^2^ were estimated using single imputed data with bootstrap

### Between-center variation in outcome

**Fig. 2:**
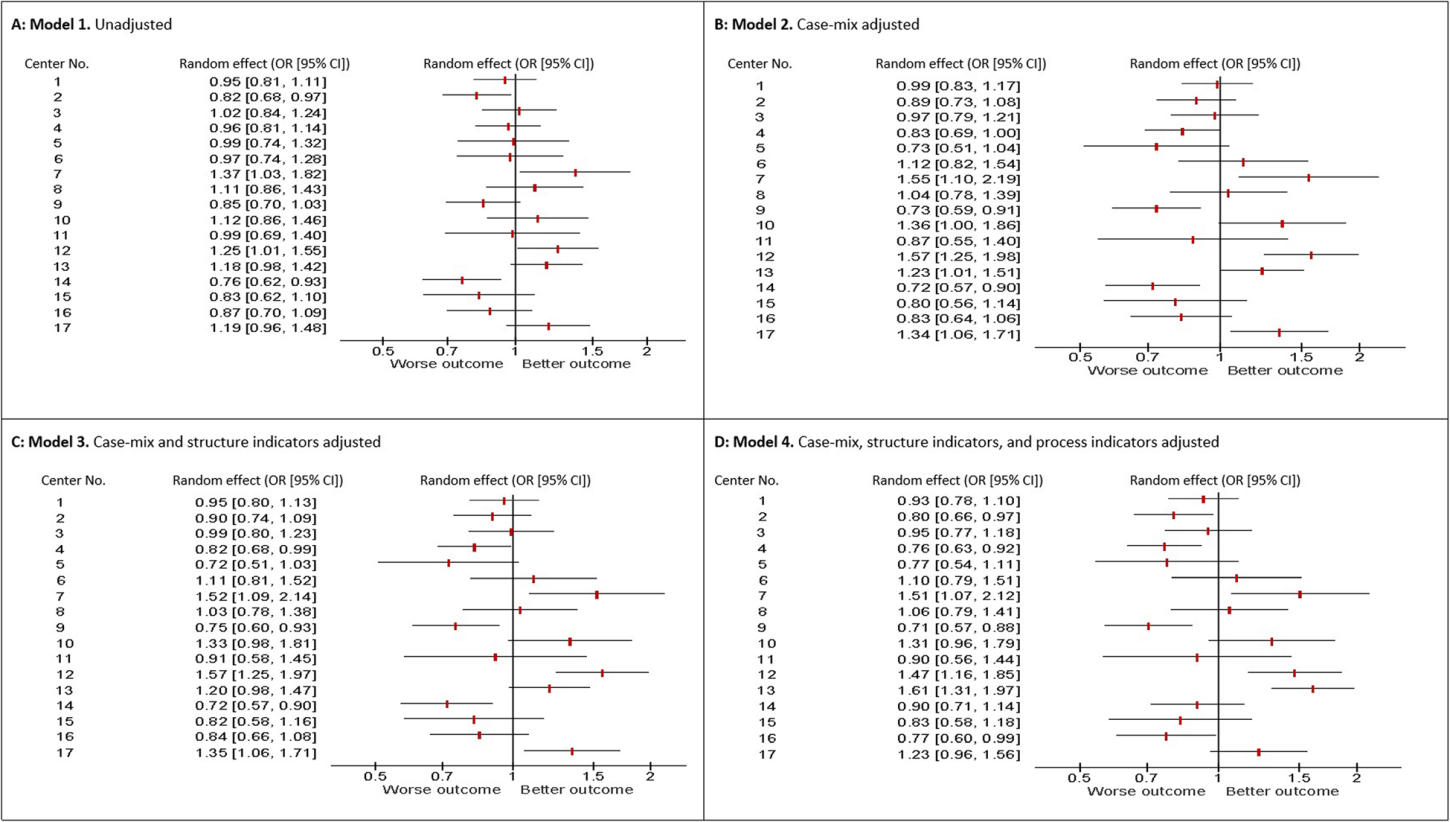
Forest plots reporting random center effect (odds ratios and 95% confidence intervals) on inverse of modified Rankin Scale at 90 days in four models using random effect proportional odds regression analysis. **a:** Model 1 (unadjusted model); **b:** Model 2 (case-mix adjusted model); **c:** Model 3 (case-mix and structure indicators adjusted model); **d:** Model 4 (case-mix, structure and process indicators adjusted model)

In model 1, which only adjusts for random variation, the amount of the between-center variation in outcome (*tau*^*2*^) was 0.040 (Table 3). The *tau*^2^ represents the amount of variability in outcome between centers. This estimate more than doubled after adding case-mix indicators (model 2, *tau*^2^: 0.086), while adding structure indicators (model 3) and process indicators (model 4) left it almost unaffected (*tau*^2^: 0.081 and 0.089, respectively). This indicates that only a small amount of between-center variation was explained by variation in performance on structure and process indicators. This finding is also reflected in the forest plots (Fig. 2), which for each model show the estimated effects of all 17 centers on the likelihood of favorable outcome. Between-center variation increased particularly after case-mix indicators were added (compare Fig. 2b with Fig. 2a), while variation remained rather constant after adding structure (Fig. 2c) and process (Fig. 2d) indicators.

## Discussion

This study focused on assessing variation in outcome between centers treating ischemic stroke with EVT in the Netherlands, and the impact of differences in case-mix and in performance on structure and process indicators. Our results show that differences in case-mix have a much larger impact on between-center differences in outcome for stroke patients treated with EVT than differences in structure and processes of care across centers. Therefore, an (unadjusted) measure of functional outcome may not be a valid indicator for quality of care when comparing stroke centers.

### Outcome indicators as measures of quality of care

Benchmarking initiatives increasingly use outcome measures to compare quality of care between centers. However, as found in this study, significant differences in performance on evidence- and consensus-based measures of (processes of) care may not be reflected in between-center differences in outcome. An important explanation for this is the observational nature of the data that are typically used in such benchmarking initiatives. When assessing the effect of interventions or processes of care on outcome in such data, confounding by indication is a major issue. For example, physicians might treat more severely affected patients faster than less severely affected patients [32, 33]. As disease severity is likely to influence outcome, in observational data it can confound the estimated relation with outcome insofar severity is insufficiently accounted for. Although we adjusted for disease severity in several ways using measured case-mix variables, we cannot preclude the possibility that our estimated effect of time to treatment on outcome is to some extent biased. This could partly be because of a residual unmeasured case-mix effect, as well as unaccounted for technical parameters. Knowing the association of center-level outcomes with process indicators and structural characteristics allows decision-makers to understand the determinants of performance and implement improvement strategies; in other words, all indicators should be retained to get an empirical (i.e., context specific) evaluation of the quality of care. If a center is an “outlying provider” even after accounting for patient case mix and process/structural characteristics, in-depth analyses and audit activities should be put in place to understand the reasons for this center’s outlying performance. Given that detecting a beneficial effect of effective interventions on outcome can be difficult even in large methodologically sound randomized trials, observing effects of evidence- and consensus-based indicators of (processes of) care on between-center differences in outcome may be even harder in benchmarking initiatives using observational registry data. In other words, when observational data are used for benchmarking, which in practice is typically the case, good performance on structure and process indicators may not be reflected in favorable outcome due to unmeasured confounding factors. Therefore, identifying and benchmarking performance on indicators with a proven contribution to favorable outcomes should be an important future direction for stroke care quality assessment and improvement initiatives.

### Between-center differences in outcome after case-mix adjustment

The observed difference of the unadjusted and adjusted estimated center effect is because of statistical consideration and is a result of both imbalance and stratification [34, 35]. Good outcomes will generally be more difficult to achieve for patients who are more severely affected at baseline. Therefore, the observed outcomes of centers with a relatively ‘severe’ case-mix will on average be less favorable (i.e. biased downwards) as compared to those of centers with a relatively ‘mild’ case-mix (i.e. biased upwards). In general, adjusting for the imbalance in case-mix would then reduce observed variation in outcome between centers. However, in this study an opposite pattern is observed. After adjusting for case-mix, actual between-center differences in outcome become visible. Apparently, centers with a more severe case-mix tend to have relatively good outcomes and vice versa. Although counterintuitive, this still underlines the necessity of appropriate case-mix adjustment in benchmarking quality performance using observational data. Besides the effect of adjusting for the imbalance, more extreme center effect after adjustment could be a result of the stratification effect. Although “stratification” usually refers to conditioning on categoric subgroups (e.g. on sex), we also use this term when continuous variables are involved, for example, age. Adjustment will generally increase standard errors, and the stratification (adjustment) effect will lead to more extreme effect estimates [34].

### Strengths and limitations

In previous studies [6, 7], a dichotomized version of the mRS was used (mRS ≥3 was considered as ‘poor outcome’) and analyzed using binary logistic regression models. In order to exploit the full ordinal nature of the mRS score, we used proportional odds regression analysis. In addition, the use of random effect analyses allowed us to estimate (the variance of) center outcomes adjusted for random variation and various other factors (i.e. case-mix, structure, and process indicators).

A first limitation of this study is the unavailability of other potentially contributing factors to between-center outcome differences, e.g. unmeasured patient characteristics, care processes, and center characteristics. A second limitation is that missing values may have introduced some bias, although we believe to have mitigated this issue considerably using multiple imputation, which is the preferred method over complete case analysis [36, 37]. A third limitation is low number of second-level units, with resulting lack of precision in *tau*^2^ estimates. A final limitation is that we only analyzed one outcome. Although the mRS is used as an outcome in virtually all modern stroke trials, our conclusions might have been different if we had used other outcomes that are relevant to stroke patients treated with EVT, like patient-reported outcomes such as quality of life. In addition, even though the mRS is an appropriate tool for assessing patient disability after stroke care, it may not be easily transferrable to outcomes research. After all, in many countries other than the Netherlands, the mRS is not routinely collected for all acute stroke patients. However, if we would have used clinical outcomes that are commonly registered in administrative databases, such as short-term mortality or readmission, we would have less certainty about our estimations because of the much smaller number of events in the context of acute stroke treatment. Moreover, from a clinical perspective these outcome measures are far less relevant in the stroke context compared to the mRS. Therefore, we used the mRS as the most powerful and clinically relevant outcome for our study.

## Conclusions

In this study, we have demonstrated that between-center differences in performance on structure and process indicators have a small impact on functional outcome of ischemic stroke patients treated with EVT, while differences in case-mix affects this variation substantially. Thus, outcome indicators may not be valid and useful for comparing and improving the quality of stroke care based on observational data. Since variation in performance on structure and process indicators captures real quality improvement potential, these indicators should be used in future benchmarking initiatives. This is especially true when a strong association exists between those indicators and outcome, as is the case for time to treatment in ischemic stroke.

## Supplementary Information

**Additional file 1: Table S1.** Case-mix characteristics of patients treated in intervention centers in the MR CLEAN Registry. **Table S2.** Quality of care indicators characteristics of the intervention centers in the MR CLEAN Registry database. **Figure S1.** Specialized EVT centers in the Netherlands. Adapted from MR CLEAN Registry study (https://www.mrclean-trial.org/mr-clean-registry/participating-centers.html). **Figure S2.** Flowchart of patient selection in the MR CLEAN Registry. **Figure S3** Forest plot reporting odds ratios with 95% confidence intervals of the fixed effects of the case-mix variables on the inverse of the modified Rankin Scale at 90 days using random effect proportional odds regression analysis in the case-mix adjusted model.

## Data Availability

The dataset analyzed during the current study is available from the MR CLEAN Registry upon request as a study proposal. Requests should be directed to the executive committee (mrclean@erasmusmc.nl). Detailed analytic methods and study materials, including log files of statistical analyses, will be made available to other researchers on request to the corresponding author (m.amini@erasmusmc.nl).

## Abbreviations

EVT: Endovascular treatment
ICA: Internal carotid artery
ICA-T: Internal carotid artery terminus
M1/M2: Middle cerebral artery
A1/A2: Anterior cerebral artery
CTA: Computed tomography angiography
mRS: Modified Rankin Scale
NIHSS: National Institute of Health Stroke Scale
ED: Emergency department
AIC: Akaike’s Information Criterion
IQR: Interquartile range
PAD: Peripheral arterial disease
MI: Myocardial infarction
HCL: Hypercholesterolemia
