# Supplementary material for: Improving quality of stroke care through benchmarking center performance: why focusing on outcomes is not enough
Source: BMC Health Serv Res. 2020 Oct 31;20:998. doi: 10.1186/s12913-020-05841-y (PMC7603730; doi:10.1186/s12913-020-05841-y)
Supplement: Supplementary file 1 — Additional file 1: Table S1. Case-mix characteristics of patients treated in intervention centers in the MR CLEAN Registry. Table S2. Quality of care indicators characteristics of the intervention centers in the MR CLEAN Registry database. Figure S1. Specialized EVT centers in the Netherlands. Adapted from MR CLEAN Registry study (https://www.mrclean-trial.org/mr-clean-registry/participating-centers.html). Figure S2. Flowchart of patient selection in the MR CLEAN Registry. Figure S3 Forest plot reporting odds ratios with 95% confidence intervals of the fixed effects of the case-mix variables on the inverse of the modified Rankin Scale at 90 days using random effect proportional odds regression analysis in the case-mix adjusted model. [file 12913_2020_5841_MOESM1_ESM.pdf]

SUPPLEMENTARY INFORMATION FOR:

IMPROVING QUALITY OF STROKE CARE THROUGH BENCHMARKING CENTER PERFORMANCE: WHY FOCUSING ON OUTCOMES IS NOT ENOUGH

Supplementary Table 1 Case-mix characteristics of patients treated in intervention centers in the MR CLEAN Registry.

N (%) / Median (interquartile range)																				
**Hospitals**	**1**	**2**	**3**	**4**	**5**	**6**	**7**	**8**	**9**	**10**	**11**	**12**	**13**	**14**	**15**	**16**	**17**	**Total**	**Missing (N)**	***P-value****
**Age**	70 (59-78)	74 (60-81)	73 (65-82)	70 (59-80)	68 (55-78)	77 (63-84)	71 (63-79)	72 (62-81)	71 (58-79)	73 (61-80)	75 (67-85)	75 (65-81)	70 (62-80)	72 (60-80)	73 (62-84)	73 (62-80)	72 (62-81)	3279	0	0.001
**Men**	204 (52)	176 (55)	117 (48)	217 (54)	33 (49)	46 (48)	42 (53)	74 (54)	107 (49)	49 (44)	9 (39)	112 (51)	157 (53)	131 (54)	27 (45)	94(53)	101 (53)	1696/3279	0	0.790
**Medical History**																				
**Previous Stroke**	63 (16)	82 (26)	28 (11)	56 (14)	8 (12)	19 (20)	14 (18)	23 (17)	38 (18)	27 (25)	0	33 (16)	41 (14)	42 (17)	14 (23)	19 (11)	39 (21)	546/3252	27	<0.001
**Atrial Fibrillation**	91 (24)	107 (34)	55 (23)	86 (21)	11 (17)	35 (37)	19 (24)	31 (23)	38 (17)	22 (20)	3 (13)	59 (29)	66 (22)	41 (17)	17 (28)	37 (21)	54 (30)	772/3236	43	<0.001
**MI**	46 (12)	56 (18)	37 (15)	56 (14)	9 (14)	14 (15)	13 (17)	11 (8)	32 (15)	16 (16)	1 (4)	31 (15)	27 (9)	37 (16)	9 (15)	25 (14)	33 (18)	453/3212	67	0.16
**PAD**	17 (4)	68 (22)	24 (10)	32 (8)	5 (8)	10 (11)	8 (10)	5 (4)	14 (7)	8 (8)	3 (15)	18 (9)	25 (9)	13 (6)	12 (20)	18 (10)	21 (12)	301/3211	68	<0.001
**Hypertension**	166 (44)	189 (59)	136 (57)	207 (52)	29 (44)	53 (56)	33 (45)	67 (50)	111 (51)	56 (54)	14 (61)	117 (56)	121 (41)	124 (53)	34 (57)	119 (67)	112 (62)	1688/3213	66	<0.001
**Diabetes**	58 (15)	57 (18)	31 (13)	67 (17)	10 (15)	17 (18)	15 (19)	20 (15)	54 (25)	16 (15)	3 (13)	44 (21)	42 (14)	35 (15)	10 (17)	31 (18)	22 (12)	532/3255	24	0.09
**HCL**	57 (15)	152 (50)	74 (35)	89 (22)	16 (24)	23 (24)	16 (23)	41 (30)	65 (30)	38 (37)	6 (27)	74 (38)	56 (20)	97 (41)	29 (48)	61 (35)	73 (43)	967/3136	143	<0.001
**pre-stroke mRS**																			72	<0.001
**0**	279 (72)	203 (64)	151 (62)	305 (77)	58 (87)	60 (63)	53 (78)	96 (70)	123 (56)	67 (66)	14 (61)	155 (72)	224 (77)	98 (45)	32 (53)	122 (68)	130 (69)	2170/3207		
**1**	59 (15)	47 (15)	47 (19)	34 (9)	4 (6)	11 (12)	2 (3)	13 (10)	40 (18)	13 (13)	4 (17)	23 (11)	30 (10)	34 (16)	9 (15)	22 (12)	32 (17)	424/3207		
**2**	22 (6)	21 (7)	22 (9)	34 (9)	1 (2)	9 (10)	0 (0)	12 (9)	20 (9)	8 (8)	0	9 (4)	16 (6)	34 (16)	4 (7)	12 (7)	17 (9)	241/3207		
**≥3**	27 (7)	47 (15)	25 (10)	25 (6)	4 (6)	15 (16)	13 (19)	16 (12)	35 (16)	13 (13)	5 (22)	27 (13)	21 (7)	51 (24)	15 (25)	23 (13)	10 (5)	372/3207		
**Location of occlusion**																			160	<0.001
**Intracranial ICA**	17 (5)	19 (6)	7 (3)	25 (6)	1 (2)	8 (9)	3 (4)	12 (10)	15 (7)	7 (7)	0	5 (2)	19 (7)	6 (3)	2 (3)	9 (5)	6 (3)	161/3119		
**ICA-T**	86 (23)	77 (25)	35 (15)	77 (20)	16 (26)	8 (9)	12 (15)	25 (20)	44 (22)	19 (18)	6 (26)	56 (27)	72 (26)	40 (17)	13 (22)	42 (25)	35 (20)	663/3119		
**M1**	219 (60)	168 (54)	153 (65)	226 (58)	36 (59)	45 (50)	54 (69)	57 (45)	106 (52)	64 (62)	16 (70)	130 (62)	166 (59)	137 (59)	37 (63)	101 (59)	100 (57)	1815/3119		
**M2**	41 (11)	45 (15)	41 (17)	61 (16)	8 (13)	28 (31)	6 (8)	31 (25)	35 (17)	12 (12)	1 (4)	19 (9)	23 (8)	43 (19)	7 (12)	19 (11)	35 (20)	455/3119		
**Other**	4 (1)	2 (1)	1 (0,4)	2 (1)	0	1 (1)	3 (4)	1 (1)	4 (2)	2 (2)	0	0	0	5 (2)	0	0	0	25/3119		
**Collaterals**																			207	<0.001
**0 (Absent)**	23 (6)	28 (9)	16 (7)	15 (4)	1 (2)	6 (7)	4 (5)	7 (6)	4 (2)	5 (5)	0	12 (6)	23 (8)	14 (6)	4 (7)	13 (8)	12 (7)	187/3072		
**1 (<50%)**	137 (38)	91 (30)	68 (30)	138 (36)	19 (32)	37 (41)	23 (30)	37 (31)	49 (24)	43 (41)	4 (17)	82 (40)	127 (46)	76 (33)	23 (39)	78 (45)	68 (41)	1100/3072		
**2 (>50% - <100%)**	153 (43)	110 (36)	89 (39)	147 (38)	25 (42)	39 (43)	33 (43)	59 (49)	75 (37)	39 (38)	14 (61)	78 (38)	91 (33)	96 (42)	20 (34)	64 (37)	58 (35)	1190/3072		
**3 (100%)**	47 (13)	78 (25)	54 (24)	89 (23)	15 (25)	9 (10)	17 (22)	18 (15)	74 (37)	17 (16)	5 (22)	34 (17)	36 (13)	45 (20)	12 (20)	18 (10)	27 (16)	595/3072		
**NIHSS score**	17 (13-20)	16 (12-20)	15 (10-18)	15 (9-18)	17 (12-20)	17 (11-21)	14 (9-18)	13 (8-18)	15 (11-20)	17 (13-21)	14 (11-16)	17 (13-20)	16 (12-19)	15 (10-20)	17 (11-21)	15 (11-20)	16 (11-20)	3224	55	<0.001
**Transferred patients from other hospital**	271 (70)	200 (62)	166 (68)	310 (77)	1 (2)	13 (14)	43 (54)	25 (18)	40 (18)	53 (47)	0	133 (62)	188 (64)	141 (58)	10 (17)	81 (45)	108 (57)	1783/3279	0	<0.001
**Time from onset to arrival in ED**	145 (80-195)	152 (78-210)	155 (120-204)	160(116-215)	53 (38-89)	75 (37-135)	120 (50-191)	68 (38-158)	89 (44-194)	141 (72-209)	52 (33-95)	126 (88-175)	154(105-200)	127 (70-166)	59 (42-142)	118 (47-186)	125 (72-188)	3155	124	<0.001
**Predicted probability of good functional outcome (%) #**	40 (21-59)	34 (14-59)	44 (24-62)	45 (24-66)	48 (32-73)	39 (14-59)	45 (27-63)	50 (24-70)	43 (18-68)	45 (19-67)	17 (9-61)	38 (15-61)	39 (23-58)	40 (21-69)	31 (11-57)	39 (18-63)	46 (21-63)	2550	729	0.004

MI Myocardial Infarction; **PAD** Peripheral Arterial Disease; **HCL** hypercholesterolemia; **ICA-T** internal carotid artery terminus; **mRS** modified Rankin Scale; **M1/M2** middle cerebral artery; **NIHSS** National Institutes of Health Stroke Scale; **IVT** intravenous thrombolysis; **ED** Emergency department.

* P-value is based on comparison between 17 centers using a non-parametric Kruskal Wallis test or Pearson's chi-square statistic.

# Predicted probability of good functional outcome (modified Rankin Scale score 0-2 at 90 days) is based on an individual-level logistic regression model predicting good functional outcome from the case-mix variables included in this table.

Supplementary Table 2 Quality of care indicators characteristics of the intervention centers in the MR CLEAN Registry database

	N (%) / Median (interquartile range)									
**Hospitals**	**1**	**2**	**3**	**4**	**5**	**6**	**7**	**8**	**9**	**10**
**Structure**										
**Hospital volume**	390	321	246	405	67	95	80	137	218	112
**Process**										
**Time from arrival in ED to groin puncture in non-transferred patients (min)**	93 (70-119)	74 (62-90)	83 (66-110)	85 (69-111)	102 (80-138)	96 (78-143)	109 (83-130)	100 (71-127)	94 (70-125)	87 (67-123)
**Time from arrival in ED to groin puncture in transferred patients (min)**	35 (25-53)	42 (28-58)	38 (27-55)	37 (25-53)	NA	21 (16-37)	30 (22-38)	31 (25-60)	43 (28-66)	37 (29-55)
**Use of general anesthesia**	87 (23)	12 (4)	45 (19)	24 (7)	19 (29)	1 (1)	7 (9)	39 (30)	4 (2)	15 (14)
**Outcome**										
**modified Rankin Scale (mRS) at 90 days**										
**mRS 0 (No symptoms)**	19 (5)	12 (4)	24 (10)	29 (8)	3 (5)	10 (11)	14 (18)	13 (11)	3 (1)	8 (8)
**mRS 1 (No significant disability)**	51 (14)	39 (13)	35 (14)	47 (14)	12 (18)	19 (20)	16 (21)	16 (14)	18 (8)	18 (19)
**mRS 2 (Slight disability)**	74 (20)	67(22)	35 (14)	54 (16)	12 (18)	9 (10)	8 (11)	22 (19)	54 (25)	19 (20)
**mRS 3 (Moderate disability)**	47 (13)	42 (14)	33 (14)	55 (16)	8 (12)	9 (10)	15 (20)	19 (17)	30 (14)	14 (14)
**mRS 4 (Moderately severe disability)**	54 (15)	25 (8)	40 (16)	40 (12)	10 (15)	8 (9)	5 (7)	8 (7)	34 (16)	9 (9)
**mRS 5 (Severe disability)**	28 (8)	17 (5)	12 (5)	22 (6)	2 (3)	5 (5)	2 (3)	6 (5)	24 (11)	4 (4)
**mRS 6 (Dead)**	97 (26)	110 (35)	65 (27)	99 (29)	19 (29)	34 (36)	16 (21)	31 (27)	53 (25)	25 (26)

ED Emergency department**; NA** not applicable, because of only one patient transferred in center 5 and no transferred patients in center 11.
* Data on number of patients per year were not shown to keep centers anonymous.

Supplementary Table 2 Quality of care indicators characteristics of the intervention centers in the MR CLEAN Registry database (continued).

	N (%) / Median (interquartile range)									
**Hospitals**	**11**	**12**	**13**	**14**	**15**	**16**	**17**	**Total**	**Missing**	***P-value*** [#]
**Structure**										
**Hospital volume***	23	217	296	244	60	179	189	3279	-	-
**Process**										
**Time from arrival in ED to groin puncture in non-transferred patients (min)**	104 (81-122)	75 (64-93)	125 (97-153)	90 (77-115)	107 (85-127)	79 (58-101)	79 (64-94)	1292	204	<0.001
**Time from arrival in ED to groin puncture in transferred patients (min)**	NA	39 (25-60)	50 (40-63)	60 (40-86)	33 (19-46)	20 (15-35)	25 (18-33)	1722	61	<0.001
**Use of general anesthesia**	0	2 (1)	285 (97)	216 (99)	8 (14)	10 (7)	4 (2)	778/3082	197	<0.001
**Outcome**										
**modified Rankin Scale (mRS) at 90 days**									214	<0.001
**mRS 0 (No symptoms)**	2 (9)	22 (10)	15 (6)	2 (1)	1 (2)	6 (4)	26 (14)	209/3065		
**mRS 1 (No significant disability)**	6 (26)	56 (26)	59 (22)	15 (7)	4 (7)	27 (16)	33 (18)	471/3065		
**mRS 2 (Slight disability)**	1 (4)	29 (14)	64 (23)	58 (29)	13 (22)	28 (17)	14 (8)	561/3065		
**mRS 3 (Moderate disability)**	1 (4)	13 (6)	33 (12)	20 (10)	3 (5)	28 (17)	34 (18)	404/3065		
**mRS 4 (Moderately severe disability)**	4 (17)	24 (11)	21 (8)	24 (12)	18 (31)	16 (10)	26 (14)	366/3065		
**mRS 5 (Severe disability)**	1 (4)	9 (4)	4 (2)	13 (6)	2 (3)	7 (4)	10 (5)	168/3065		
**mRS 6 (Dead)**	8 (35)	61 (29)	79 (29)	71 (35)	18 (31)	57 (34)	43 (23)	886/3065		

ED Emergency department**; EVT** endovascular treatment; **eTICI** extended Thrombolysis in Cerebral Infarction (eTICI) score, ranging from 0 (no reperfusion) to 3 (full reperfusion).
NA not applicable, because of only one patient transferred in center 5 and no transferred patients in center 11.
* Data on number of patients per year were not shown to keep centers anonymous.
[#] *P-value* is based on comparison between 17 centers using a non-parametric Kruskal Wallis test or Pearson' s chi-square statistics.

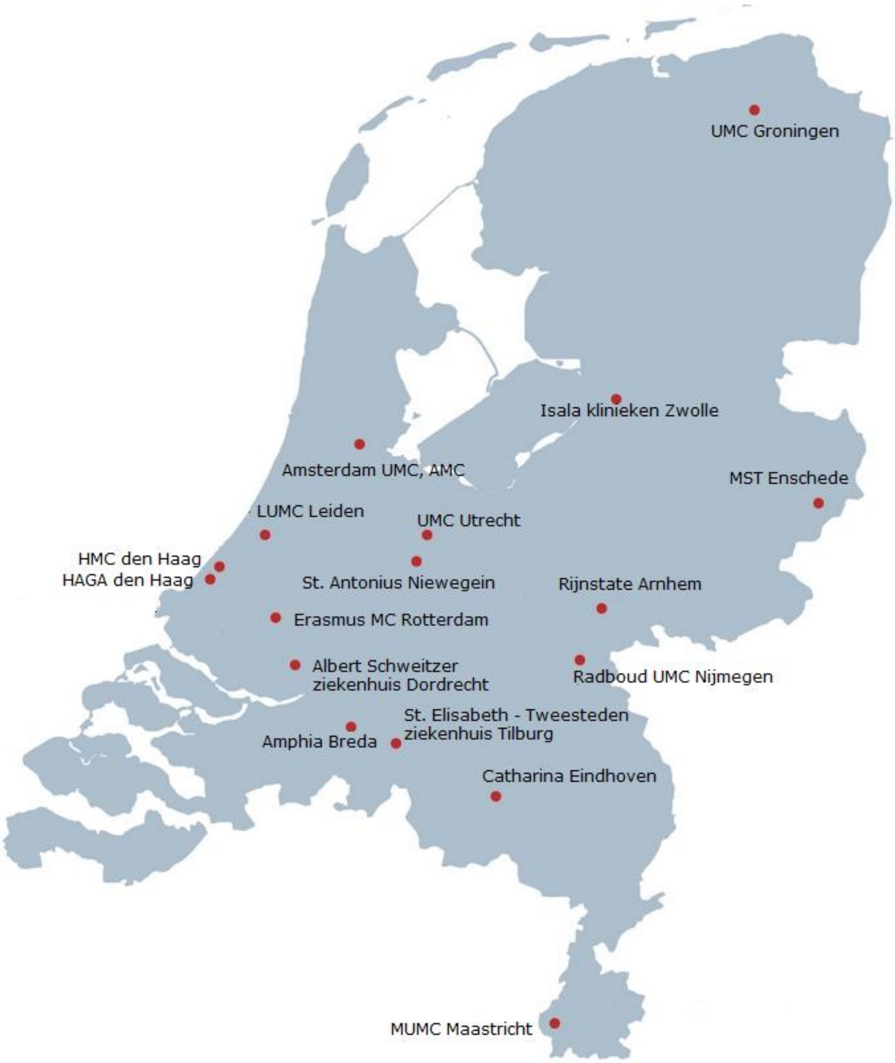

Supplementary figure 1. Specialized EVT centers in the Netherlands. Adapted from MR CLEAN Registry study (https://www.mrclean-trial.org/mr-clean-registry/participating-centers.html).

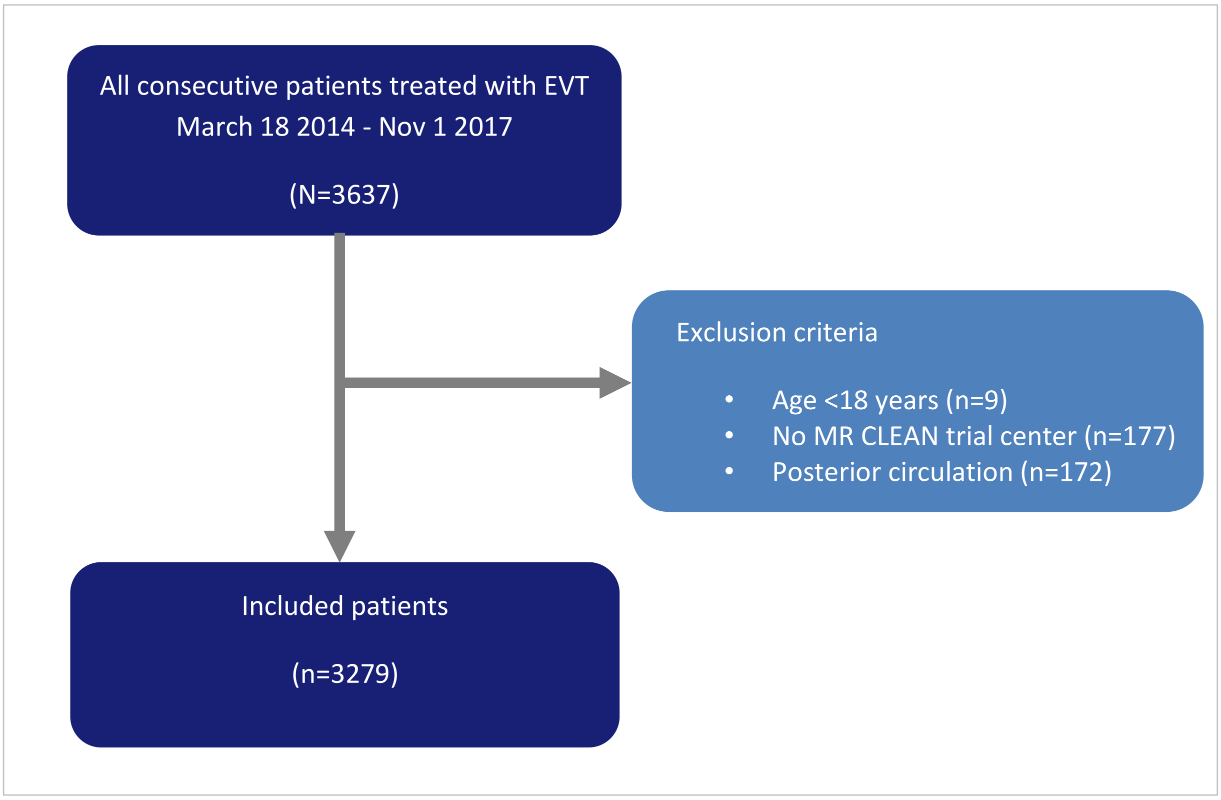

Supplementary Figure 2. Flowchart of patient selection in the MR CLEAN Registry.

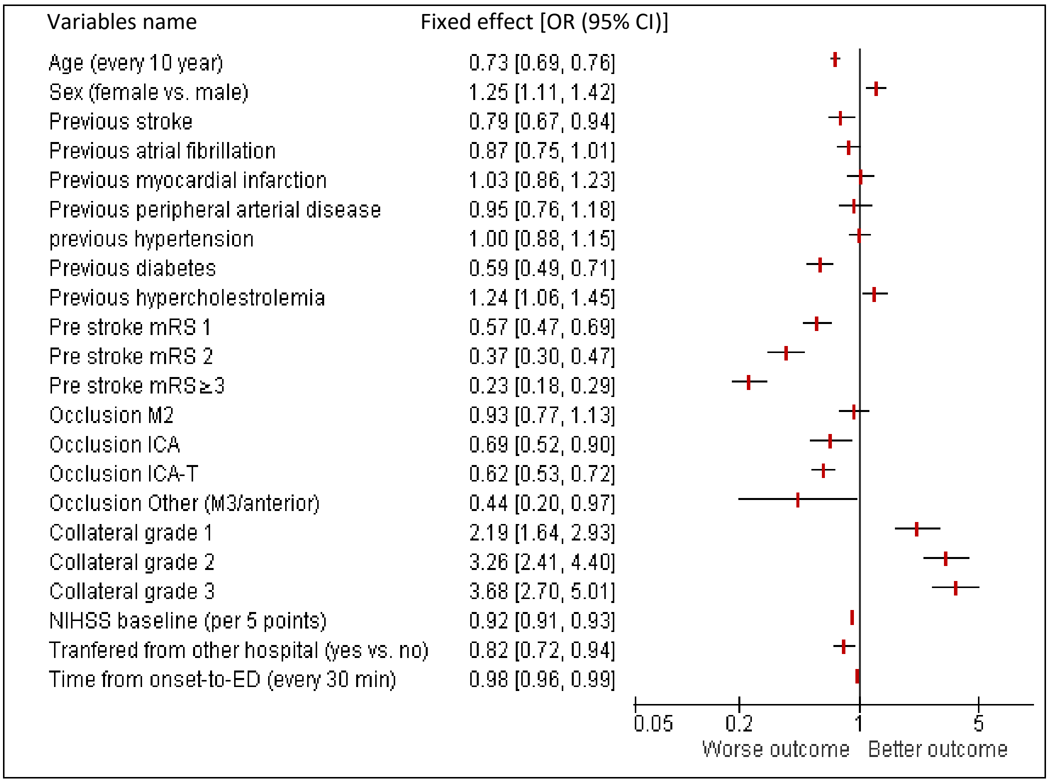

Supplementary Figure 3 Forest plot reporting odds ratios with 95% confidence intervals of the fixed effects of the case-mix variables on the inverse of the modified Rankin Scale at 90 days using random effect proportional odds regression analysis in the case-mix adjusted model.

The reference categories are: pre stroke mRS 0 for pre stroke mRS, occlusion M1 for occlusion location and collateral grade 0 for collateral grade.

mRS modified Rankin Scale; **ICA-T** internal carotid artery terminus; **M1/M2** middle cerebral artery 1/2; **NIHSS** National Institutes of Health Stroke Scale; **ED** emergency department.
